# A sustainable approach to enhance heavy hydrocarbons removal in landfarming treatment

**DOI:** 10.1007/s10532-023-10025-6

**Published:** 2023-03-25

**Authors:** Camilla Di Marcantonio, Agostina Chiavola, Alessandra Noce, Elisabetta Straccamore, Andrea Giannuzzi, Jacopo Jirillo, Francesco Gallo, Maria Rosaria Boni

**Affiliations:** 1 Department of Civil, Building and Environmental Engineering (DICEA), Sapienza University of Rome, Rome, Italy; 2 ITELYUM Regeneration S.p.A., Ceccano, Italy

**Keywords:** Biodegradation, Biostimulation, Bioaugmentation, Industrial sludge, Circular economy, Petroleum hydrocarbons

## Abstract

The present study aimed to evaluate the best strategy to enhance the degradation rate of heavy petroleum hydrocarbons (HPH) contaminated soil in a landfarming plant. Samples of real contaminated soil, further spiked with HPH, were treated in mesocosm reactors simulating the landfarming system. One reactor was operated without any modification compared to the real landfarming plant. The other three reactors were operated with different strategies to improve the removal rate: biostimulation (BS) through the addition of nitrogen and phosphorus; bioaugmentation (BA) with the inoculation of sludge produced in the treatment of the process water from the oil re-fining plant of the same industrial area; combination of biostimulation and bioaugmentation (BAS). The biostimulation (BS) was the most effective strategy, leading to a reduction of the remediation time by 35% as compared to the traditional treatment. Bioaugmentation (BA) also provided positive effects leading to a reduction of the remediation time by 24%; its performance improved further when the addition of sludge was combined with the increase of phosphorous (BAS). Therefore, the key tool was represented by the phosphorous availability, whereas the application of sludge was most useful to provide waste with a new possibility of reuse, thus fulfilling the principles of the circular economy. The final characterization showed that the treated soil was suitable for reuse in industrial areas according to the legislation in force.

**Supplementary Information:**

The online version contains supplementary material available at 10.1007/s10532-023-10025-6.

## Introduction

One of the ambitious Horizon Europe objectives is to ensure that 75% of soils are healthy by 2030. However, according to the European Mission Board for Soil health and food (2020), 60–70% of soils in Europe are still in an unhealthy state. To address the challenging goal posed by Horizon Europe, implementation of sustainable and effective remediation strategies for contaminated soils must be strongly fostered (European Commission 2021). Petroleum hydrocarbons are among the most commonly found hazardous pollutants in contaminated soils (Haleyur et al. 2019). Due to their biodegradable nature, some of them can be removed by bioremediation, through the activity of microbial population either autochthonous and/or added from an external source. When properly designed and operated, the process is capable of transforming hazardous organic pollutants into stable and not harmful products, such as CO_2_, CH_4_, H_2_O and new biomass (Yuniati 2018). Concerning the physicochemical and thermal technologies, the biological processes are considered more environmentally friendly and less expensive while ensuring a high remediation efficiency (Ossai et al. 2020). The biological treatments can be applied both in-situ and ex-situ.

Landfarming represents one of the most commonly applied treatments for ex-situ bioremediation due to its relatively simple implementation and operation (Lukić et al. 2017). This technology involves the spreading of a thin layer of contaminated soil (50–60 cm) on a treatment area, where it is being aerated or turned over to ensure aerobic conditions inside. Indeed, biodegradation of petroleum hydrocarbons is mostly due to aerobic reactions carried out by heterotrophic bacteria, which require optimal conditions for their activity (United States Environmental Protection Agency 2017). It is possible to stimulate the removal process by adding nutrients or amendments (referred to as biostimulation), or inoculating the soil with microbial species which can be either selected or not specific for the contaminants (referred to as bioaugmentation) (Speight and Arjoon 2012; Qin et al. 2013). Many scientific studies demonstrated the possibility to enhance the biodegrading capacity of the indigenous microbial community by the addition of nutrients (Taccari et al. 2012; Grace Liu et al. 2013; Abed et al. 2014; Bidja Abena et al. 2019). Usually, nitrogen and phosphorous are dosed through inorganic amendments, such as ammonium sulphate, potassium dihydrogen phosphate or phosphoric acid.

Nutrients could be also supplied in contaminated environmental matrices through the addition of amendments such as compost, sewage sludge, fresh soil and other organic waste materials which are rich in nitrogen and phosphorous (Wang et al. 2016; Wu et al. 2017; Chikere et al. 2019). These amendments can be also used as a primary microbial substrate fostering cometabolic processes; besides, they can represent a further source of microorganisms, thus promoting biostimulation effects (Alves et al. 2019; Chaudhary and Kim 2019). The most studied bioaugmentation practice consists of the addition of pure or mixed culture of petroleum-degrading bacteria (Grace Liu et al. 2011, 2013; Wu et al. 2016; Haleyur et al. 2019; Zhou et al. 2019). Nonetheless, a non-selected consortium of microorganisms present in waste materials can represent a more feasible option for real-scale applications being easier to obtain and to manage and more adaptable to the complex environmental conditions of contamination (Fernández-Luqueño et al. 2016; Lukić et al. 2017). Additionally, the employment of waste material accomplishes circular economy principles since a new option of reuse is provided instead of its final disposal. This practice allows also to save the costs related to waste material disposal and of purchasing selected microbial consortia. A good example of this practice is reported in the study conducted by Alves et al. (2019). They investigated the feasibility of an aerobic bioremediation process, i.e. composting, for the treatment of the solid phase of hydrocarbon residues by applying two different co-substrates, fish sludge and municipal sewage sludge, which showed a bioaugmentation potential due to their rich diversity in the microbial biomass. Ros et al. (2010) pointed out that the addition of organic amendments, particularly sewage sludge, can be considered as a useful strategy for improving the effectiveness of landfarming biodegradation processes in hydrocarbon polluted soils, since it increased the hydrocarbon degradation rate as compared to the unamended soil. This was explained as a consequence of the increase in bacterial and fungal populations and also the content of phosphorous and nitrogen nutrients as a consequence of the use of sewage sludge as amendment. Additionally, Wang et al. (2016) were able to enhance the removal of light and heavy petroleum hydrocarbons from polluted soils by a landfarming process, simulated in microcosms, dosed with activated sludge from an oil-refining wastewater facility, compost, selected total petroleum hydrocarbon-degrading bacteria and fern chips. Interestingly, the best performances were obtained when oil-refining sludge and compost were added. The application of industrial sludge, as proposed by the latter study, seems to represent an interesting strategy to enhance the efficiency of landfarming. However, it has not been extensively studied so far and therefore needs further investigations.

The present experimental activity focused on the bioremediation of a soil contaminated by heavy non-aromatic hydrocarbons (C > C20) in a full-scale landfarming treatment in operation since 2000 at a re-fining industrial plant. Since the removal rate was very low and unable to achieve in a reasonable time the target set by the Italian legislation on contaminated soil (i.e. 750 mg/kg SS as total hydrocarbons), then it was decided to investigate if biostimulation and/or bioaugmentation strategies were capable of enhancing the bioremediation process.

The present study aims at evaluating an experimental procedure to enhance the removal efficiency of the landfarming process applied to a hydrocarbon-contaminated soil of an industrial plant. Three different strategies were tested, i.e.: (1) biostimulation, (2) bioaugmentation and (3) combination of biostimulation and bioaugmentation. In the (2) and (3) cases, the sludge from the wastewater treatment plant of the same industry was used as inoculum. Therefore, the internal reuse of resources is fostered also in the soil remediation field, in compliance with the principles of circular economy.

As far as the authors know, the present study represents the first attempt of reusing the sludge for remediation activity within the same industrial plant. Furthermore, the three different strategies were compared and the main influencing operative parameters monitored in order to highlight the best was to optimize the remediation process. The results obtained were considered of relevant importance by the company managing the plant for a further implementation of the treatment at full-scale.

## Materials and methods

### Soil and sludge sampling and characterization

Samples of soil were collected from an industrial area located in central Italy (Frosinone province). The soil in this area is contaminated by heavy hydrocarbons, thus according to the rules set by the Italian legislation a remediation process is on-going, specifically a landfarming treatment.

The soil was sampled before remediation and it was characterized for its main physical, chemical and microbiological properties. Its texture was recognized to be of sandy-gravel type (gravel 64% and sand 36%), according to the Shepard-modified classification (Schlee 1973). The mean characteristics resulted to be as follows on dry weight (dw) basis: 7.9 pH, 1.4% total organic carbon content (TOC), 450 mg/kg total nitrogen (N_tot_), < 0.1 mg/kg total phosphorous (P_tot_), 701,000 CFU/g bacterial counts (BC), 3100 mg/kg total petroleum hydrocarbons (TPH) consisting of mainly heavy petroleum hydrocarbons (HPH, C > C12) and light petroleum hydrocarbons (LPH: C ≤ C12) < 0.1 mg/kg. After collection, the soil was immediately used to fill the experimental reactors.

The biological inoculum used for the bioaugmentation experimental tests consisted of samples of the excess sludge from the treatment of the process water coming from the re-fining plant located in the same industrial area which converts waste oils into regenerated lubricant bases. The treatment of process water is carried out through the following main steps: stripping and evaporation, activated sludge process for the condensate, clarification to separate sludge from treated effluent, and finally excess sludge thickening (final sludge concentration of about 4%). The sludge extracted from the thickening unit was characterized based on the European Council Directive 86/278/EEC consolidated in 2022 on the protection of soil when sewage sludge is used in agriculture (a summary of the characterization was reported in Table 1 S.M.). It was shown that it did not exceed the limits posed by the directive and therefore it was considered suitable for the experimental objectives (European Council 2022).

Additionally, the main bacterial strains present in the sludge were identified through DNA sequencing. For this purpose, sample dilution was seeded by inclusion on a Petri dish with specific medium (membrane faecal coliform agar). After incubation at 44 °C for 24 h, the colonies were isolated on non-specific Nutrient agar medium. The two most representative colonies were selected by morphological evaluation and genomic DNA was extracted following the instruction of NZY Microbial gDNA Isolation kit by NZYtech Lda (a spin column silica-based system). From individual DNA extracts, specific regions of the 16S ribosomial DNA gene and of the gyrase B gene were amplified using the universal primers Fn3/Fn1 and GyrB-UP-1F/GyrB-UP-2R, respectively (Yamada et al. 1999; Barghouthi 2011). The amplicons were sequenced by the Sanger method and the resulting sequences were analyzed using Blastn, searcing in the Nucleotide database of the NCBI (https://blast.ncbi.nlm.nih.gov/Blast.cgi).

For the first colony, the best alignment was *Bacillus thuringiensis* with 100% Query Cover and 100% Identity but it could not be discriminated from the closely-related species *B. cereus* that showed 100% Query Cover and 100% Identity also (Daffonchio et al. 2006). For the second colony, the best alignment showed a 100% Query Cover and 87% Identity; since this value is below 97%, that is commonly chosen as threshold for species level, the colony was identified only at genus level as *Acinetobacter species* (details about the sequence were reported in Table 2 S.M.)*.* The former species is common in the activated sludge; the latter species can be related to the presence of hydrocarbon in the treated wastewater and it was found to be active on hydrocarbons removal by Das and Chandran (2011) and Rodriguez-Campos et al. (2019).

### Spiking procedure

Since the values originally detected in the soil sample did not represent a severe condition for biodegradation (i.e. 3100 mg/kg dw), it was decided to spike it to increase the concentration of total petroleum hydrocarbons (TPH) up to the value of about 35,000 mg/kg dw of TPH, which falls within the range proposed by the United States Environmental Protection Agency (2017) for landfarming treatment. The applied spiking procedure is based on Chiavola et al. (2010) and modified according to the recommendation of Northcott and Jones (2000). Specifically, 150 mL of a petroleum lubricant base (100% long-chain hydrocarbons (C20 < C < C50) and absence of polycyclic aromatic hydrocarbons) was added to 4.35 kg dw of soil sample. The base had the same hydrocarbon composition as the non-spiked soil. After the spike, the soil was carefully mixed and then air-dried under a fume hood for 24 h. The reactors were finally filled with the spiked soil, covered with aluminium foils to avoid photodegradation and left in a dark room for 8 weeks, as suggested by several authors (Brinch et al. 2002; Couling et al. 2010). At the end of this period, the TPH were measured and found to be equal to 33,000 ± 1980 mg/kg dw, as average among three replicates.

### Experimental setup

The experimental reactors were tailor-made designed for this study, in order to reproduce the same conditions and characteristics of the landfarming treatment carried out at the full-scale. The reactors consisted of 316 grade stainless-steel tanks (15 × 30 × 20 cm^3^), with a base sloped by about 1% in order to facilitate collection and extraction by means of a tap positioned at the bottom of any leachate produced in the treatment. Every tank was equipped with a manually operated irrigation system fed by tap water. Irrigation frequency was controlled in order to maintain the value of the Volumetric Water Content (VWC) within the range 40–70%. The VWC was measured by a FieldScout TDR 100 Soil Moisture Meter. The environmental temperature was weekly monitored by a standard probe (21 °C as average). Every 3–4 days, the soil was aerated by a manual turning over, as done in the full-scale treatment. Each reactor was filled with 5 kg of soil previously spiked as described in "Spiking procedure" section.

Three different strategies were applied to the soil to evaluate their capability in enhancing the efficiency of the landfarming treatment to remove TPH, i.e. biostimulation, bioaugmentation and combination of biostimulation and bioaugmentation. Particularly, the following 4 series of tests were carried out, each one in duplicate:

*C*—which simulated the full-scale landfarming treatment without the application of any enhancing strategy, and used as a control for comparison with the other conditions.

*BS*—which simulated the biostimulation process, carried out by the periodical addition of a synthetic nutrient solution to stimulate the activity of the microorganisms already present in the soil (autochthonous). Specifically, 0.38 gP/kg dw were added every 3 weeks through a phosphoric acid solution at 60 g/L H_3_PO_4_. Furthermore, 0.92 gN/kg dw were provided through a urea solution at 100 g/L CH_4_N_2_O only at the beginning of the experimental activity since the total nitrogen content measured in the soil was always enough to ensure the optimal ratio of C/N/P equal to 100/10/1 (Metcalf & Eddy 2015; Wu et al. 2016; United States Environmental Protection Agency 2017).

BA—which simulated the bioaugmentation process, carried out by the addition of sludge every 5 weeks as inoculum and amendment (i.e. t = 0 d, 5 d, 10 d, 5 d). The sludge was collected from the storage tank after thickening as described in "soil and sludge sampling and characterization" section, and immediately added to the reactors. The dosage of sludge was initially selected based on the Italian legislation, which sets the maximum amount of waste sludge for reuse in agricultural applications equal to 5 ton dw/hectare. Based on the dimensions of the experimental reactors, the latter amount corresponded to 0.4 L of sludge added at t = 0 d. Concerning this value, no relevant increase of the biodegradation rate was observed for the first weeks of experiments. Additionally, the former dosage can be considered very precautionary for a soil which is destined to be reused in an industrial area where no limits are settled by the legislation. For these two reasons, the dosage of sludge was increased to 13 ton dw/hectare, which corresponded to 0.2 L at t = 5, 10 and 15 d.

*BAS*—which combined the biostimulation and bioaugmentation, in order to exploit any possible synergistic effect. Therefore, the same conditions as applied in *BA* and *BS* were applied also to the *BAS* reactors, in terms of quantity and frequency of nutrient and sludge addition.

The experimental activity started after 8 weeks from the time of filling the reactors. At this time (i.e. t = 0 d), solutions and/or sludge, depending on the type of test, were added for the first time. Every week, the VWC values were measured in each reactor and water was added if needed; then, the soil was always mixed and sampled (for about 80 g) from different points and depths in each reactor. The samples were analysed by an external laboratory and all the analytical methods were certified by the unique Italian Accreditation Body, named Accredia, which ensured the reliability and high-quality standards of the results. The measured parameters were the following: pH, TOC, N_tot_, P_tot_, aerobic CFU (AeBC), anaerobic CFU (AnBC), HPH and LPH. In Supplementary materials, Table 3 S.M., were listed the methods followed for the analytical determinations, the corresponding limits of quantifications and more details about the methods (Italian Ministry for Agricultural Policy 1999, 2002; US EPA 2012). Particularly, for HPH and LPH determination, the following EPA methods were applied: EPA 3550C 2007 + EPA 8015C 2007 and EPA 5021A 2014 + EPA 8015C 2007, respectively. The limits of quantification for the latter analytes were: 5 mg/kg dw and 1 mg/kg dw for HPH and LPH, respectively (US EPA 1996).

At the end of the experimental time (t_e_ = 112 days), the soil was characterized in order to assess any risk related to its reuse as a filling material in the industrial area, according to the Italian law regulating for soil and rock excavation (DPR 120/2006). Particularly, it was characterized by determining all the parameters foreseen by the Italian Code on the Environment (Testo Unico Ambientale, D.Lgs. 152/2006) for soil in industrial area. Leaching tests were also performed with the aim to evaluate if the treated soil could be considered as non-hazardous waste and therefore it could be reused under safe conditions. The list of parameters with their concentration are reported in Supplementary materials (Tables 4, 5 S.M.).

### Calculation methods

The percentage removal efficiency (R) achieved by each enhancement strategy was calculated as below:

1 $$R \left[ \% \right] = \frac{{C_{0} - C_{t} }}{{C_{o} }}{ } \cdot 100$$

where C_o_ and C_t_ stand for the concentrations of hydrocarbons measured at the beginning of the test (t = 0) and at any sampling time (t), respectively.

A statistical analysis of the data was performed to obtain further insight into the relationship among the different parameters and factors monitored during the experimental activity.

Particularly, the Spearman correlation coefficient was calculated among the TPH removal efficiency and the other parameters. The test was selected since it is suitable to evaluate relationships involving qualitative and quantitative ordered variables in the case of partially linear correlation (Conover 1999). The calculation of the correlation coefficient and the correlation test, which is necessary to validate the statistical significance of the obtained coefficients, was performed by applying the R package “Hmisc” (R Core Team 2021).

The whole data set of all the parameters analyzed during the experiment was processed also through the Principal Component Analysis (PCA) by applying the R package “FactoMineR” (Lê et al. 2008). The results of the PCA were tested through pairwise PERMANOVA which allows for the assessment of the statistical differences between the groups of treatments. The analysis was performed by applying a function of the R package “RVAideMemoire”, considering the Euclidean distance matrix and 1000 permutations (Hervé 2022). The difference between groups was statistically significant if p-value was below 0.05.

The concentrations of TPH measured in each reactor at any sampling time were modelled by applying the linearized form of the zeroth, first, second orders and saturation kinetic models, according to the equations reported by Alexander (1994) and Boni et al. (2018). Moreover, the Half-Life (t_1/2_) and time necessary to reach the target concentration set by the Italian legislation on contaminated soil, equal to 750 mg/kg dw of TPH, were also calculated based on the best fitting model identified for each test.

## Results and discussions

### TPH removal

**Fig. 1:**
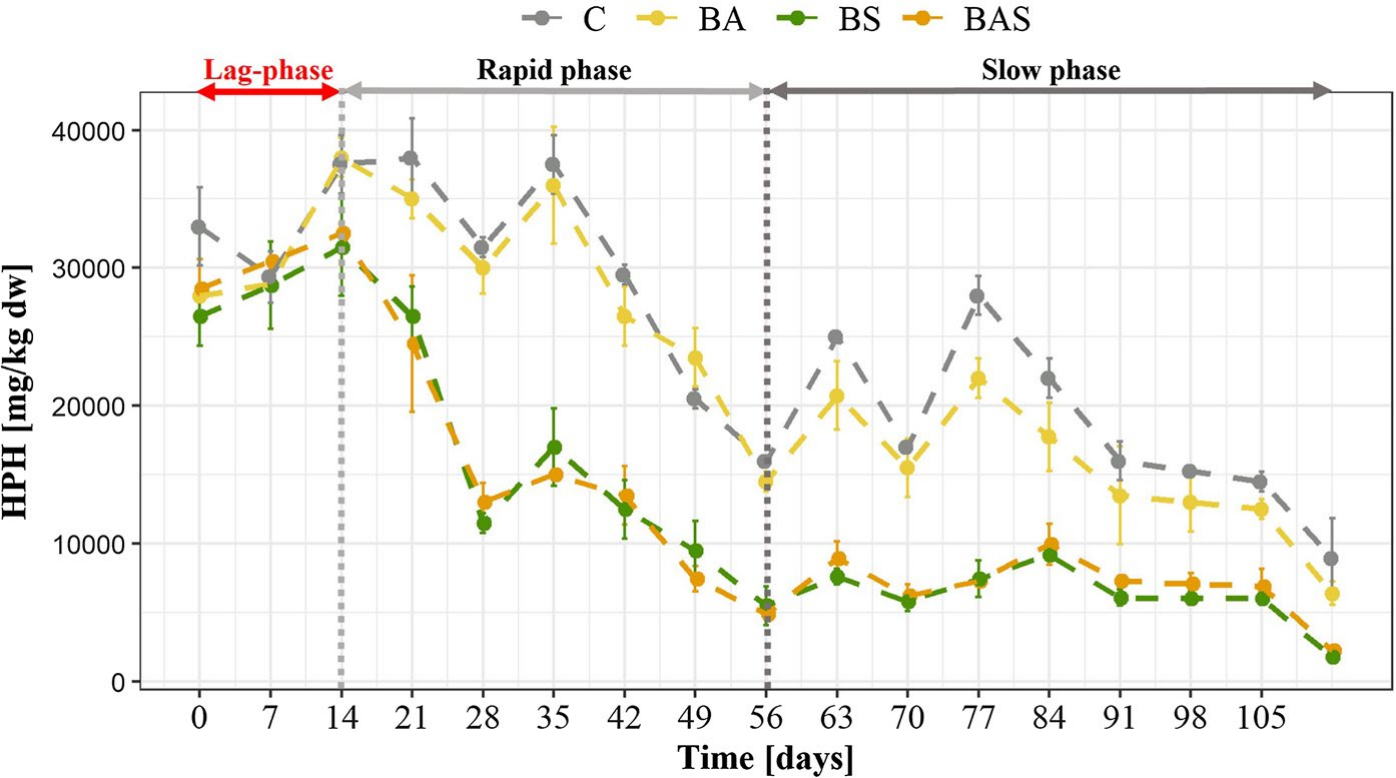
Average concentration time-profile of HPH in the experimental reactors. The error bars indicate the standard deviation among test duplicates

Fig. 1 shows the average concentration of HPH measured during the entire duration of the tests. The concentrations of LPH were always found to be below the detection limits in all the samples.

It can be observed that the HPH removal pattern was characterized by three well distinct phases under all the experimental conditions: Lag-phase, Rapid phase and Slow phase, as reported in the upper part of the figure. The Lag-phase corresponded to the first fourteen days of the experiment: this period was the time needed by microorganisms after the eight weeks of stabilization to progressively adapt to the new experimental/environmental conditions due to the addition of water, nutrients or sludge according to the experimental conditions (C, BA, BS and BAS, respectively). As explained by Metcalf & Eddy (2015), this is a time required for the microorganisms to acclimatise to new conditions such as changes in oxygenation, salinity, pH or temperature. Additionally, an enzyme induction might have occurred before a significant cell division and biomass production could occur. In this phase, chemical reactions, thermodynamic and biological processes may also undergo a latency period prior to reaching their maximum rate (Hamill et al. 2020).

The next phase, identified between the fourteenth and fifty-sixth day of experimentation, and reported as a Rapid Phase, was characterized by the maximum reaction rate in each reactor. It was related to the fast removal of the most easily biodegradable fraction of HPH, as will be discussed below (paragraph 3.11).

The final phase reported as a Slow phase, between day fifty-sixth and the end of the experiment, was characterized by a slowing down of the removal kinetics.

A similar trend, characterized by a rapid and slow phase, was also identified by other authors in the biodegradation of hydrocarbons in long-term polluted soils (Thiele-Bruhn and Brümmer 2005). Moreover, Wang et al. (2016) also observed that the decay trend of TPH by landfarming under different conditions flatted off after a period of faster decay.

Differences in the time-profile of hydrocarbons in the distinct tests are here discussed; particularly, the removal trends observed in the control test (C) and in the bioaugmentation test (BA), were very similar. The same can be assessed for the trends highlighted in the biostimulation (BS) test and in the combination of biostimulation and bioaugmentation (BAS). Furthermore, in both C and BA tests, data were affected by a wider variability, particularly during the slow phase. Differently, the trends in BS and BAS seem to reflect a more stable removal process. The final average concentrations of HPH and the corresponding removal efficiency were as follows, for C, BA, BS and BAS, respectively: 8950 mg/kg dw, 6400 mg/kg dw, 1800 mg/kg dw, 2250 mg/kg dw, and 69%, 78%, 93% and 92% removal.

These results highlight that the best remediation process occurred in BS and BAS tests. The small increase in the removal efficiency in the BAS treatment can be considered not significant compared to BS, as proved by the PERMANOVA results reported in Table 6 S.M.. Biostimulation is working with or without the presence of the inoculum. So Bioaugmentation is not working effectively in contrast to biostimulation which is really the most efficient process. The key to enhancing landfarming remediation efficiency under the tested conditions is represented by the phosphorous concentration.

It is noteworthy that the addition of the sludge as inoculum became effective in enhancing the removal rate only when nutrients were also added (BAS test); in this case, the removal efficiency was similar to that measured in the BS test. Therefore, the key to enhancing the landfarming remediation efficiency under these conditions is represented by the phosphorous content increase.

Comparing the C and BA tests shows that the addition of waste sludge (bioaugmentation) provided an enhancement of the remediation process compared to the traditional treatment (C) carried out with the addition neither of sludge nor of nutrients. Specifically, the removal efficiency increased by 9% in the case of BA compared to C. The difference was found to be significant by the application of PERMANOVA, as reported in Table 6 S.M.

#### Chromatograms analysis

The analysis of the chromatograms was carried out to support the hypothesis that during the Rapid phase the degradation of the easier biodegradable HPH was carried out. Figure 1 S.M. shows the comparison, on the retention time basis, among the chromatograms of samples collected at the beginning of the experiment (t0), at the end of the Rapid phase (t8) and at the end of Slow phase (t16) in tests C, BS and BAS. The reliability of the comparison between the retention times was ensured by the perfect overlap of the first peak (4.9 min), which represents the internal standard injected into each sample. The analysis was carried out looking at the position (retention time) of the most abundant fraction of hydrocarbons; instead, the intensity of the peaks was not evaluated since the sample preparation was modified during the experiment, especially by varying the dilution factor (due to the high variation of HPH concentration during the remediation process). Observing the first figure (Fig. 1 S.M_C) related to the control (reactor C), it is evident that the chromatograms of the three samples are perfectly overlapped, which indicates that the retention time of the most abundant fraction of the unresolved complex mixture (UCM), the so-called central value, remained around 9.6 min. It proves that the most abundant hydrocarbon fraction is always the same throughout the experiment. In BS and BAS, the retention time of the area related to the UCM always ranged from 7 to 11 min, but there was a change in the shape of those areas, which corresponds to a shift of the central values of the retention time. This change indicates a change in the most abundant fraction of hydrocarbons (ISPRA and ARPA-APPA, 2011). During the Rapid phase in BS and BAS, there was a shift of the central value towards shorter times, which indicates a relative increase of shorter chain hydrocarbons, suggesting the breakdown of the main molecules. On the other hand, in the Slow phase, the peak of UCM shifted to the right, indicating that in the final phase of the treatment the shorter chains of hydrocarbons, produced in the Rapid phase, were degraded; furthermore, the residual most abundant fraction was characterized by a longer chain of HPH as compared to the fraction predominant at the beginning of the experiment. Finally, the chromatograms analysis proved that there was no significant difference between BS and BAS, confirming that even in terms of characteristics of hydrocarbon UCM, the two operating conditions were comparable Other studies also observed similar biodegradation pathways in landfarming systems: the rapid biodegradation of the lower weight hydrocarbons (with a shorter retention time in the chromatographic analysis) determined a progressive increase of the relative abundance of hydrocarbons having higher molecular weights (with a longer retention time) compared to the total content (Savin et al. 2015; Wang et al. 2016).

#### Principal component analysis

**Fig. 2:**
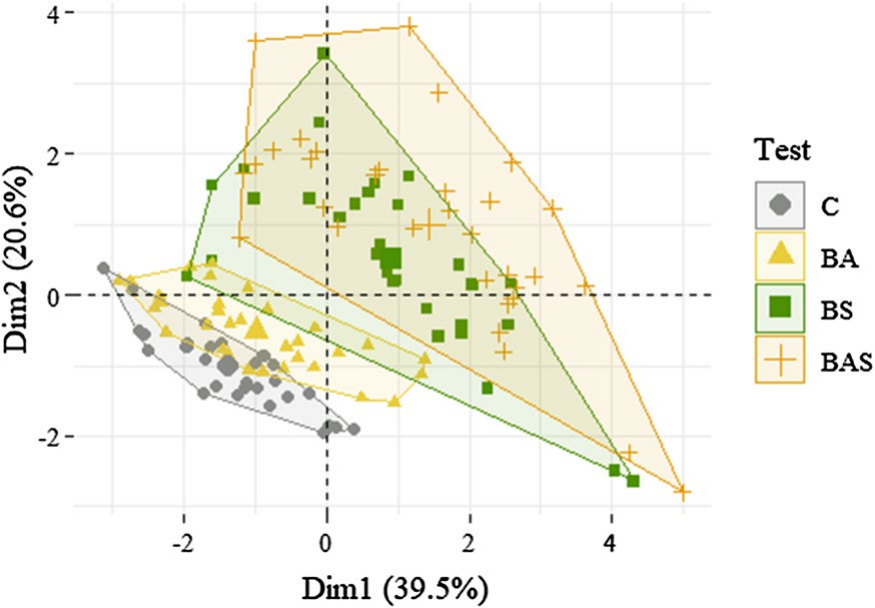
Principal component analysis: individuals plot

The principal component analysis (PCA) was applied to the data to evaluate the overall effect of the different strategies considering all the parameters monitored. Through the PCA, the number of measured variables was reduced to two principal components, which explains most of the variance of the date, i.e. the 56.5%. The results of the analysis are reported in Fig. 2 as individuals, i.e. each symbol represents a sample considering all the monitored parameters; they were coloured based on the Test.

It is possible to observe that the samples collected from C and BA tests are mainly located in the III quadrant, while those from BS and BAS tests are more dispersed in the rest of the plot. This confirms the similarity of the characteristics of the samples taken from C and BA, and from BS and BAS. The application of the PCA statistically demonstrated that the addition of sludge changed, but only slightly, the characteristics and progress of the landfarming treatment. It was further confirmed that the combination of biostimulation and bioaugmentation produced comparable effects as biostimulation alone. These evaluations were confirmed by the application of the pairwise PERMANOVA which indicates a significant difference between all the treatments with the only exception of BAS and BS, i.e. p-value > 0.05 (results were reported in Table 6 S.M.).

### Factors influencing TPH removal process

**Fig. 3:**
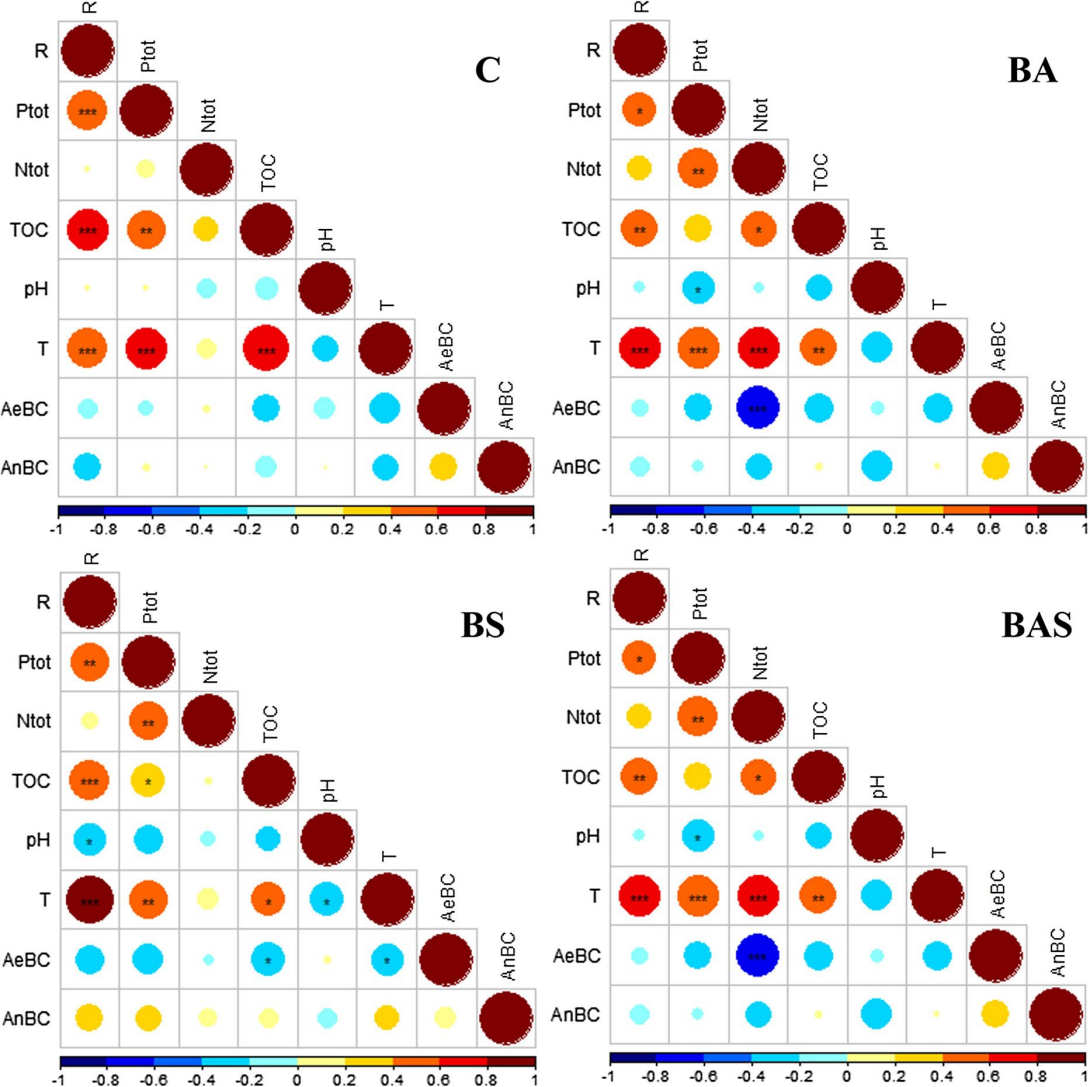
Correlation matrices. The colours of the circles represent the value of the Spearman correlation coefficient as reported in the legend. The symbols into the circles represents the statistical significance of the coefficients

The influence of the parameters monitored during the experiments was assessed through the Spearman correlation coefficient. This coefficient is suitable for ordered variables; partially linear correlation; non-normal distributed and homoscedastic data verified through the application of the Shapiro and Levene tests, respectively (data not here shown) (Conover 1999). The correlation matrices are presented in Fig. 3, considering the four different experimental conditions separately. The statistical significance of each coefficient was also verified through the so call Correlation test; the results are reported in the figure as different symbols depending on the p-value: * for < 0.05, ** for < 0.01, *** < 0.001 and empty for no significance.

Observing the four matrices, the removal of TPH (R) was well correlated with total phosphorous (P_tot_), total organic carbon (TOC) and temperature (T), with the following characteristics: they are direct correlations; they are valid under all the experimental conditions; they show the highest coefficients (above 0.4) and they are statistically significant. Interesting information is obtained on the role of the nutrients: P_tot_ seems to be the key factor for the TPH removal process, whereas total nitrogen (N_tot_) is not correlated with R, proving that this nutrient is not a limiting parameter for the degradation process.

The time-profiles of the concentrations of the parameters discussed in this paragraph (P_tot_, T, TOC, N_tot_, AeBC, AnBC and pH) are reported in Supplementary materials (Figs. 2, 3 S.M.).

To better elucidate the role of P_tot_ in the degradation process, the specific consumption rate of phosphorous was calculated. Specifically, based on the amount added to BS and BAS (380 mg P_tot_ every three weeks) and the residual measured at the end of the Rapid and Slow phase, the average P_tot_ consumed in the same time interval was firstly calculated: 342 mg and 340 mg for BS and BAS in the Rapid phase, respectively, and 337 mg and 334 mg for BS and BAS in the Slow phase, respectively. It can be noted that the amounts of phosphorous consumed in BS and BAS were very similar. Then, the specific consumption rate of phosphorous resulted to be: 0.05 mgP_tot_/mgTPH and 0.04 mgP_tot_/mgTPH for BA and BS in the Rapid phase, respectively, and 0.11 mgP_tot_/mgTPH and 0.12 mgP_tot_/mgTPH for BA and BS in the Slow phase, respectively. Since the removal rate of hydrocarbons was lower in the Slow phase, the specific phosphorus consumption decreased as a consequence. Furthermore, the specific consumption values were also similar for the BS and BAS tests.

Considering TOC measured in each sample, it was calculated the C:P ratio during the Rapid and Slow phases for both BS and BAS. In the first phase, the value resulted to be 100:5 and 100:4, respectively, which is slightly above the value recommended for optimal biodegradation (i.e. C:P equal to 100:1) (United States Environmental Protection Agency 2017; Wu et al. 2019). In the Slow phase, the ratio resulted to be 100:11 and 100:12 for BS and BAS respectively, which is one order of magnitude higher than the recommended value. The hypothesis is that microorganisms exerted an extraordinary phosphorous demand to be able to metabolize the most recalcitrant fraction of hydrocarbons. However, no other evidence was found in the scientific literature to confirm such a hypothesis. Leys et al. (2005) observed that high concentrations of N and P could slow down the biotransformation of polycyclic aromatic hydrocarbon due to the high increments in the salinity. Similarly, Oje Obinna et al. (2015) found an initial rise of available phosphorus during biodegradation of crude oil in the soil, which was attributed to the ability of microorganisms to solubilize phosphate in the soil. As the concentration of phosphorus in the environment increased, feedback inhibition of phosphatase activity was observed due to phosphate, which inactivated further phosphorus solubilisation and as a consequence its availability for bioremediation.

About the other monitored parameters, the pH values showed a wide range of variability: from 6.4 and 8.8 in all the reactors. However, these values still fall within the range defined as favourable to biodegradation (United States Environmental Protection Agency 2017). This variability may be a natural consequence of the processes since no buffers or other adjustments to pH were applied to constrain the value of this parameter. The Rapid phase was characterized by an average pH of 8.1 ± 0.1, whereas in the following phase it slightly dropped reaching 7.7 ± 0.1 in the C and BA reactors and 7.2 ± 0.1 in BS and BAS. This trend can be related to the degradation process, which was more intense in the Rapid phase in all reactors and particularly in BS and BAS. The formation of carbolic acid, as proved by Wang et al. (2016), could be a product of the degradation responsible of the pH decrease that occurred in the Slow phase. Moreover, the addition of phosphoric acid every three weeks can also be responsible for the pH reduction in the latter rectors.

The anaerobic CFU (AnBC) was two orders of magnitude lower than the aerobic CFU and resulted not being correlated with most of the other parameters. The periodic mixing of the soil maintained the soil under aerobic conditions, which were unfavourable for the development of anaerobic microorganisms. The content of AeBC did not show significant variations along the time of the experiments and therefore it could not be correlated with TPH removal. An indirect correlation was found with N_tot_ in the samples collected from the reactors where waste sludge was added (BA and BAS), which can be related to the accumulation of nitrogen due to the addition of sludge along with a reduction of its consumption by the aerobic biomass related to the slowing down of the degradation process. The aerobic CFU in BA reactor was one order of magnitude higher than in C and comparable with the content measured in BS and BAS, thus confirming the biostimulation effect due to the addition of sludge. Specifically, the average AeBC values related to the entire duration of the experiment were as follows: 2.4·10^7^, 1.5·10^8^, 6.4·10^8^, 8.2·10^8^, in C, BA, BS and BAS, respectively. In agreement with the time profile of the degradation process, the AeBC contents in BS and BAS were characterized by higher values in the Rapid phase, which gradually decreased in the Slow phase where it remained almost stationary. Furthermore, in the Rapid phase the average value of the aerobic CFU in BAS was higher than the value detected in BS (Rapid phase: 8.8·10^8^ CFU for BS and 1.1·10^9^ CFU for BAS; Slow phase: 4.0·10^8^ CFU for BS and 4.9·10^8^ CFU for BAS). Therefore, the addition of sludge positively contributed to bacterial activity due to the increase in the content of microorganisms.

### Degradation kinetics of TPH

The evaluation of the best fitting kinetic model was carried out for the two phases of the removal process. The results obtained are summarized in Table 1, where the coefficient of determination (R^2^), the kinetic constants and the half-life time (t_1/2_) are reported for each model and experimental condition. The best-fitting model was selected based on the R^2^ and coherence with the theoretical trend.

**Table 1:** Results of the kinetic model evaluation

Kinetic order	Parameters		Rapid phase				Slow phase			
			C	BA	BS	BAS	C	BA	BS	BAS
Zeroth	R^2^		*0.79*	*0.81*	0.82	0.87	*0.53*	*0.60*	0.42	0.40
	k_0_	mg kg^−1^d^−1^	*517.86*	*494.90*	558.67	593.88	*100.13*	*118.08*	83.72	85.59
	t_1/2_	d	*36*	*38*	28	27	*185*	*161*	185	190
First	R^2^		0.77	0.77	*0.85*	*0.91*	0.50	0.54	*0.46*	*0.43*
	k_1_	d^−1^	0.02	0.02	*0.04*	*0.04*	0.01	0.01	*0.02*	*0.02*
	t_1/2_	d	35	36	*19*	*17*	88	62	*34*	*39*
Second	R^2^		0.74	0.69	0.77	0.81	0.47	0.48	0.44	0.43
	k_2_	kg mg^−1^d^−1^	7.9E–07	8.2E–07	2.9E–06	3.6E–06	6.4E–07	1.1E–06	5.8E–06	4.4E–06
	t_1/2_	d	34	32	11	9	42	23	6	7
Saturation	R^2^		0.97	0.95	0.85	0.53	0.78	0.60	0.30	0.36
	ks	mg kg^−1^d^−1^	10.60	46.41	57.46	47.56	− 54.20	− 113.87	− 149.61	− 117.62
	Ka	mg kg^−1^	− 26,985	− 24,176	− 16,830	− 17,960	− 28,604	− 31,005	− 22,558	− 22,327

The best fitting model for each condition is highlighted in italics

The best-fitting model was found to be the zeroth order for C and BA and first order for BS and BAS in both phases.

The fitting was always better for the data obtained by the Rapid phase (i.e. R^2^ equal to 0.79, 0.81, 0.85 and 0.91 for C, BA, BS and BAS, respectively), whereas the R^2^ values of the Slow phase were lower (i.e. below 0.6), according to the stationary pathways of the degradation process. Moreover, the values of the kinetic constants were always higher in the Rapid phase according to the rapid degradation of TPH experimentally observed. The same trend was identified by Thiele-Bruhn and Brümmer (2005) in Polycyclic Aromatic Hydrocarbon long-term polluted field soil.

The zeroth-order reaction rate is independent of the concentration of the contaminant; besides, it represents a process where the initial substrate concentration is much higher than the half-saturation constant. The first-order kinetic is directly dependent on the substrate concentration; therefore, the rate of removal decreases as the available substrate to degrade becomes less available (Alexander 1994).

The analysis of the kinetics constants confirmed the comparable performances of BS and BAS, since the values of the kinetic constants for both processes were the same.

A simulation of the time required to reach the target concentration set by the Italian legislation for contaminated soil (t_t_), (equal to 750 mg/kg dw of TPH), was finally performed. The t_t_ values were obtained by applying the best fitting model and the values of the kinetic parameters of the Slow phase for each experimental condition since it represents the rate-limiting phase of the entire remediation process. For this purpose, it was assumed that the initial concentration was equal to the concentration measured at time = 56 d, which was considered approximately the beginning of the Slow phase. The t_t_ resulted equal to 152 d, 116 d, 105 d and 99 d for C, BA, BAS and BS, respectively. The biostimulation is confirmed to be the best strategy to enhance the remediation rate: under the present experimental conditions and starting from a very severe level of contamination, the time required to bring the concentration below the limit set by the law in force was reduced by 53 d due to the addition of phosphorous (BS) and by 36 d due to the addition of sludge (BA).

### Risk assessment for treated soil reuse

At the end of the experimentation, the treated soil was characterized with the aim to evaluate if there might be a potential risk due to its reuse as filling material in the same industrial area where it was collected. Particularly, the concentration of 51 analytes was measured on the soil; furthermore, this was subjected to a leaching test and then 25 analytes were measured in the leachate.

All the parameters measured on the soil resulted to be below the limits posed by the Italian legislation in force (data reported in Table 4 S.M and Table 5 S.M.). Differently, some parameters in the leachate were measured at values above the limits: indeed, average Chemical Oxygen Demand (COD) was about 70 mg O_2_/L in all the reactors versus a limit set to 30 mg O_2_/L; average nitrates in reactors BS and BAS were 101 and 170 mg/L NO_3_^−^, respectively, versus a limit set to 50 mg NO_3_^−^/L. The high COD concentration can be related to the residual concentration of TPH which was still present in the treated soil. Nitrates exceeded the limits in the reactors where nitrogen solution was added at the beginning of the experiment to stimulate the degradation process. This is something that should be considered for the scale-up of the proposed biostimulation strategies.

The results of the final characterization and the leaching test proved that the soil at the end of each test was suitable for reuse in commercial and industrial areas independently of the type of enhancement strategy applied. Furthermore, the addition of sludge did not cause negative effects on the soil quality according to the Italian legislation in force for soil and rock excavation and its reuse, as reported in "Experimental setup" section.

## Conclusions

The experimental activity carried out at laboratory scale allowed to compare the effects of three different strategies to enhance the landfarming remediation efficiency for a TPH contaminated soil: biostimulation (BS), bioaugmentation (BA) and combination of biostimulation and bioaugmentation (BAS). Concernig the control reactor (C), operated without under traditional conditions, the best improvement was achieved by biostimulation, followed by the combination of biostimulation and bioaugmentation, and then by bioaugmentation only. Indeed, the simulation carried out based on the best kinetic model showed a possible reduction of the time required to reach the standard posed by the Italian legislation equal by 35%, 31% and 24% for BS, BAS and BA compared the traditional treatment.

These results prove that the key tool to enhance landfarming remediation efficiency, under the applied conditions, is represented by phosphorous availability. However, the reuse of a waste (i.e. the sludge) instead of its disposal, even if the advantage obtained in terms of treatment performance improvement was not so high, fulfils the aims of the circular economy which nowadays must be pursued as much as possible (Wang et al. 2016). Furthermore, the reduction of the time needed to reach the required concentration of TPH imposed by the Italian legislation is of high interest for the economic affordability of the remediation process.

Finally, the risk assessment showed that the addition of this sludge did not produce any additional harm related to the possible reuse of the treated soil, according to the Italian legislation.

The step forward of the present study is to scaling-up the selected strategy to evaluate its cost-technical effectiveness under the more complex conditions of real scale.

## Supplementary Information

Below is the link to the electronic supplementary material.

Supplementary file1 (DOCX 890 kb)

## Data Availability

All data generated or analysed during this study are included in the manuscript.
