# Supplementary material for: A sustainable approach to enhance heavy hydrocarbons removal in landfarming treatment
Source: Biodegradation. 2023 Mar 25;34(5):417–30. doi: 10.1007/s10532-023-10025-6 (PMC10442250; doi:10.1007/s10532-023-10025-6)
Supplement: Supplementary file 1 — Supplementary file1 (DOCX 890 kb) [file 10532_2023_10025_MOESM1_ESM.docx]

A sustainable approach to enhance heavy hydrocarbons removal in landfarming treatment

Camilla Di Marcantonio^1,*^, Alessandra Noce^1^, Agostina Chiavola^1^, Elisabetta Straccamore^2^, Andrea Giannuzzi^2^, Jacopo Jirillo^2^, Francesco Gallo^2^, Maria Rosaria Boni^1^

^1^Sapienza University of Rome, Department of Civil, Constructional and Environmental Engineering (DICEA), Rome, Italy

^2^ ITELYUM Regeneration S.r.l., Via Monti Lepini 180 - 03023 Ceccano, Italy

*Corresponding author: Tel.: (+39) 06 44585314; E-mail address: camilla.dimarcantonio@uniroma1.it

Table 1 S.M. Sludge samples characterization.

| **Sludge addition time** | **LPH (C<= 12)** | **HPH (C>12)** | **Dry waight (105°C)** | **pH** | **TOC** | **Ntot** | **Ptot** | **Aerobic bacterial counts** | **Anaerobic bacterial counts** |
| --- | --- | --- | --- | --- | --- | --- | --- | --- | --- |
|  | mg/kg dw | mg/kg dw | % |  | % dw | mg/kg dw | mg/kg dw | UFC/g dw | UFC/g dw |
| t0 | 11 | 670 | 3.3 | 8.3 | 9.4 | 62900 | 0.1 | 4.8E+08 | 1500000 |
| t5 | 27 | 160 | 3.2 | 6.7 | 3.2 | 5650 | 0.5 | 1.6E+08 | 70000 |
| t10 | 24 | 29 | 5.9 | 8.5 | 4.5 | 3870 | 6.68 | 1.2E+08 | 240000 |
| t15 | 19 | 500 | 4 | 6.7 | 3.5 | 34500 | 1360 | 27000000 | 5100000 |
| Average | 23 | 340 | 4.1 | 7.6 | 5.15 | 26730 | 341.82 | 1.97E+08 | 1727500 |

Table 2 S.M. Bacterial speciation details.

|  | **16S rDNA sequencing** | Primer |
| --- | --- | --- |
| **Colony A** | CAGGTGGTGCATGGTTGTCGTCAGCTCGTGTCGTGAGATGTTGGGTTAAGTCCCGCAACGAGCGCAACCCTTGATCTTAGTTGCCATCATTAAGTTG | Fn3 |
|  | AGTGCCCAACTTAATGATGGCAACTAAGATCAAGGGTTGCGCTCGTTGCGGGACTTAACCCAACATCTCACGACACGAGCTGACGACAACCATGCACCACCTGTCACTCTGCTCCCGAAGGAGAAGCCCTATCTCTAGGGTTTTCAGAGGATGTCAAGACCTGGTAAGGTTCTTCGCGTTGCTTCGAATTAAACCACATGCTCCACCGCTTGTG | Rn1 |
|  | GTAAAATCCATTACCAAAAATATGAAAGAGGTATTCCGGTTGCGGATTTAAAAGTCATTGGTGACACAGATCAAACAGGAACAATAACTCGATTTAAACCAGATCCGGAAATTTTCCAAGAAACAACAGTATACGATTTTGATACGCTAGCAACTCGTATGCGTGAATTAGCGTTTTTAAATCGTAATATTAAATTAACAATTGAAGATAAACGTGAACATAAGCAAAAGAAAGAATTCCATTACGAAGGTGGAATTAAATCATACGTTGAGCATTTAAATCGCTCAAAACAACCGATTCATGAAGAGCCTGTGTACGTAGAAGGTTCAAAAGATGGTATTCAGGTTGAGGTTTCTCTTCAATATAACGAAGGATACACAAATAATATTTACT | GyrB1 |
|  | CTTCATCATTTGATAAAATCTTATCTAAGCGCGCCTTTTCCACATTAATAATTTTACCCTTCAGCGGTAAAATTGCTTGGAAATGACGATCGCGTCCTTGTTTTGCAGATCCACCCGCAGAGTCACCCTCTACGATGTAAATTTCACTAATTGCTGGATCTTTCGAAGAGCAATCAGCTAATTTACCAGGTAAACTTGAAACTTCTAACGCACTCTTTCGACGTGTCAATTCACGCGCTTTTTTCGCAGCTACACGTGCACGTGCAGCCATCGTACCTTTTTCTACAATTTTTCGCGCTACATTAGGATTTTCTAGTAAGAACTTTTCA | GyrB2 |
| **Colony B** | TACAGGTGCTGCATGGCTGTCGTCAGCTCGTGTCGTGAGATGTTGGGTTAAGTCCCGCAACGAGCGCAACCCTTTTCCTTATTTGCCAGCGGGTTAAGCCGGGAACTTTAAGGATACTGCCAGTGACAAACTGGAGGAAGGCGGGGACGACGTCAAGTCATCATGGCCCTTACGACCAGGGCTACACACGTGCTACAATGGTCGGTACAAAGGGTTGCTACCTAGCGATAGGATGCTAATCTCAAAAAGCCGATCGTAGTCCGGATTGGAGTCTGCAACTCGACTCCATGAAGTCGGAATCGCTAGTAATCGCGGATCAGAATGCCGCGGTGAATACGTTCCCGGGCCTTGTACACACCGCCCGTCACACCATGGGAGTTTGTTGCACCAGAAGTAGGTAGTCTAACCGTAAGGAGG | Fn3 |
|  | CTACCTACTTCTGGTGCAACAAACTCCCATGGTGTGACGGGCGGTGTGTACAAGGCCCGGGAACGTATTCACCGCGGCATTCTGATCCGCGATTACTAGCGATTCCGACTTCATGGAGTCGAGTTGCAGACTCCAATCCGGACTACGATCGGCTTTTTGAGATTAGCATCCTATCGCTAGGTAGCAACCCTTTGTACCGACCATTGTAGCACGTGTGTAGCCCTGGTCGTAAGGGCCATGATGACTTGACGTCGTCCCCGCCTTCCTCCAGTTTGTCACTGGCAGTATCCTTAAAGTTCCCGGCTTAACCCGCTGGCAAATAAGGAAAAGGGTTGCGCTCGTTGCGGGACTTAACCCAACATCTCACGACACGAGCTGACGACAGCCATGCAGCACCTGTAT | Rn1 |
|  | ATCCTGTCTATCCACTCAAAGTCGTGGGTGATACCGACAAAACCGGGACTACTGTTCGTTTCTGGCCAAGTGCCGAGACCTTTAGTCAAACTATTTTCAATGTTGATATTTTGGCGCGCCGTTTGCGTGAGCTTTCATTCCTGAATGCCGGTGTGCGTATTGTGCTACGTGATGAACGTATCAATGCAGAACATGTGTTTGATTATGAAGGTGGTTTGTCTGAATTCGTAAAATATATCAACCAGGGCAAAACCCATCTGAATGATATCTTTCATTTTACTGTGCAAGCAGACAATGGCATTGGTGTAGAAGTTGCCTTGCAATGGAATGACACGTATCAAGAAAACGTGCGTTAGTTTTACCAATAACATTCCACAAAAAGATGGTGGTACACATTTAGCTGGTTTCCGTGCTGCTTTAACCCGTGGCTTAAACAGCTATATGGAAAGCGAAAACTTGCTCAAAAAAGAGAAAGTTGCTGTATCGGGTGATGATGCACGTGAAGGTTTAACCGCGATTGTTTCGGTAAAAGTGCCTGATCCTAAATTCTCTTCACAAACCAAGGAAAAACTGGTTTCAAGTGAAGTGAAACCTGCTGT | GyrB1 |
|  | TCTTCACGACCAATACCACAGCCCAACGCTGTAATCAGCGTACCGACTTCCTGACTCGAGATCATCTTGTCGAAACGTGCACGTTCTACGTTCAGGATTTTACCTTTCAGTGGCAGAATGGCTTGCATCTTACGGTTACGGCCCTGTTTGGCAGAACCACCCGCAGAGTCACCTTCGACCAGGTACAATTCAGACAATGCCGGATCTTTTTCCTGACAGTCCGCCAGTTTACCTGGCAAACCGGCAATATCCAGCGCACTCTTACGACGGGTCATTTCACGCGCTTTACGTGCAGCATCCCGCGCACGTGCAGCATCGATAATTTTTCCGGCAATCGATTTTGCTGCTTGCGGATTTTCCAGCAGGTATTCCGAGAATGACTTGTTCATCGCCTGCTCTACAGCAGGTTTCACTTCACTTGAAACCAGTTTTTCCTTGGTTTGTGAAGAGAATTTAGGATCAGGCACTTTTACCGAAACAATCGCGGTTAAACCTTCACGTGCATCATCACCCGATACAGCAACTTTCTCTTTTTTGAGCAAGTTTTCGCTTTCCATATAGCTGTTTAAGCCACGGGTTAAAGCAGCACGGAAACCAGCTAAATGTGTACCACCATCTTTTTGTGGAATGTTATTGGTAAAACAACGCACGTTTTCTTGATACGTGTCATTCCATTGCAAGGCAACTTCTACACCAATGCCATTGTCTGCTTGCACAGTAAAATGAAAGATATCATTCAGATGGGTTTTGCCCTGGTTGATATATTTTACGAATTCAGACAAACCACCTTCATAATCAAACACATGTTCTGCATTGATACGTTCATCACGTAGCACAATACGCACACCGGCATTCAGGAATGAAAGCTCACGCAA | GyrB2 |

Best alignments from database search

|  | **Description** | **Max score** | **Total score** | **Query score** | **E-value** | **% Identity** | **Accession** |
| --- | --- | --- | --- | --- | --- | --- | --- |
| **Colony A** | Bacillus thuringiensis strain HER1410 chromosome, complete genome | 730 | 9436 | 100% | 0.00 | 100% | CP050183.1 |
|  | Bacillus cereus strain DLOU-Weihai chromosome, complete genome | 730 | 9431 | 100% | 0.00 | 100% | CP040342.1 |
|  | Bacillus sp. SH8-8 chromosome, complete genome | 730 | 9436 | 100% | 0.00 | 100% | CP031065.1 |
| **Colony B** | Acinetobacter sp. 185 chromosome, complete genome | 981 | 13685 | 100% | 0.00 | 86.89% | CP049916.1 |
|  | Acinetobacter chinensis strain WCHAc010005 chromosome, complete genome | 909 | 11468 | 100% | 0.00 | 85.42% | CP032134.1 |
|  | Acinetobacter johnsonii strain Acsw19 chromosome, complete genome | 902 | 11991 | 100% | 0.00 | 85.26% | CP043307.1 |

Table 3 S.M. Analytical methods with reference to the Italian legislation and the corresponding limits of quantification and details of the protocols followed for the measure of Light, heavy and total hydrocarbons and Bacterial count.

| **Analyte** | | **Method** | **Limit of quantification** | **Unit of measurement** | **References** |
| --- | --- | --- | --- | --- | --- |
| pH | pH | DM 13/09/1999 SO n° 185 GU n° 248 21/10/1999 Met III.1 DM 25/03/2002 GU n° 84 10/04/2002 | 2 | / | (Italian Ministry for Agricultural Policy, 1999) |
| Total nitrogen | Ntot | DM 13/09/1999 GU SO n° 248 21/10/1999 Met.XIV.2 | 200 | mg/kg dw |  |
| Total phosphorous | Ptot | DM 13/09/1999 SO n° 185 GU n° 248 21/10/1999 | 0.1 | mg/kg dw |  |
| Aerobic bacterial counts | AeBC | DM 08/07/2002 SO n° 156 GU n° 179 01/08/2002 Met II.1 | 10 | UFC/g dw | (Italian Ministry for Agricultural Policy, 2002) |
| Anaerobic bacterial counts | AnBC |  |  |  |  |
| Total petroleum hydrocarbons | TPH | EPA5021A 2014 +EPA8015D 2003; EPA 3550C 2007 + EPA 8015C 2007 | 5 | mg/kg dw | (EPA, 1996) |
| Light petroleum hydrocarbons (C ≤12) | LPH | EPA 5021A 2014 + EPA 8015C 2007 | 1 | mg/kg dw |  |
| Heavy petroleum hydrocarbons (C>12) | HPH | EPA 3550C 2007 + EPA 8015C 2007 | 5 | mg/kg dw |  |
| Total organic content | TOC | UNI EN 15936:2012 | 0.5 | % dw | (National Agency for Unification, 2012) |

*Bacterial counts protocol*

The method used for Microbial Charge Count is DM 08/07/2002 SO n.156 OJ n 179 01/08/2002 Met II.1. The count is performed on a plate using agar culture medium. A representative portion of the medium sample is placed in contact with the diluent solution; then the pour plate is applied to both primary suspension and subsequent dilutions (1:10 scale) on 90mm diameter Petri plate. Culture medium is added to the plates and stirred and solidified. The plates are incubated in a thermostat set at 25°C under aerobic, anaerobic or microaerophilic conditions depending on the microbial population of interest. After 7 days, the colonies grown on the various plates are counted, and according to the dilution processed the final result as CFU/g.

*Heavy hydrocarbons protocol*

*Pre-treatment*

5 g (+/-0.10) of dried sample (oven-dried at 40°C) are extracted by adding 20 mL of surrogate solution and then 5 mL of extraction solvent Hexane-Acetone (96% - 4%). The extraction is performed through 15 s of vortex, ultrasonic bath for 25 min and a final settling phase. The clarified part is than sampled and the internal standards spiked.

*Instrumental conditions*

Model: Agilent 6890 FID detector

- Injection temperature: 320°C
- Splitless injection mode
- Volume of injection: 1µL
- Column: DB-5 HT, 15m x 0.25mm, ID x 0.1μm
- Chromatographic conditions: 50°C for 3 min, 40°C per minute until 320°C for 2 min, 40°C per min until 360°C for 3 min,
- Carrier gas: helium
- Flux: constant at 1.5mL/min for 11.5min, 3mL/min until 2mL/min for 2min
- Detector FID: temperature 370°C; gas H_2_ 35-45mL/min, air 350-450ml/min, Make-up (He o N2) 20-40mL/min
- Acquired signal: FID-column compensation

Reporting limit: 5.0 mg/kg

Internal Standard: BAM-K010g Mixture of Diesel Oil and Lubricating Oil

*Light hydrocarbons protocol*

*Pre-treatment*

2.5 g (+/-0.10) of wet sample are added into 20 mL of water and 20µl surrogate solution (Clorobenzene d5), then the vial is closed by a PTFE cap and stirred through a vortex.

*Instrumental conditions*

Model: Agilent 7890A FID detector

- Injection temperature: 150°C
- Splitless injection mode (1:1)
- Volume of injection: 1µL
- Column: DB624, 30m x 0.32mm ID x 1.8 μm
- Chromatographic conditions: 45°C for 3 min, 15°C per minute until 220°C for 1 min
- Carrier gas: helium
- Flux: constant at 1.8 mL/min
- Detector FID: temperature 250°C; gas H_2_ 40mL/min, air 450ml/min, Make-up N_2_ 45mL/min
- Headspace carries: 13 psi
- Hoven temperature: 80°C
- Needle temperature: 130°C
- Transfer line temperature: 135°C

Reporting limit: 1.0 mg/kg

Internal Standard: ULTRA Scientific Gasoline-Regular, Unleaded

*Total hydrocarbons protocol*

The total hydrocarbons are determined as sum of the light and heavy fractions.

Table 4 S.M. Parameters analysed at the end of the experiment on the treated soil. The reported limits referred to the Italian D.Lgs. 152/2006: part IV, Title V, annex 5- Table 1, column B.

| **Analyte** | **Unit of measurement** | **C** | **BA** | **BS** | **BAS** | **Limits** |
| --- | --- | --- | --- | --- | --- | --- |
| pH | - | 9.05 | 8.65 | 7.5 | 6.85 |  |
| Dry waight (105°C) | % | 89.4 | 88.15 | 87.9 | 87.6 |  |
| 1,2,3-trimethylbenzene | mg/kg dw | < 1 | < 1 | < 1 | < 1 |  |
| 1,2,4-trimethylbenzene | mg/kg dw | < 1 | < 1 | < 1 | < 1 |  |
| 1,3,5-trimethylbenzene | mg/kg dw | < 1 | < 1 | < 1 | < 1 |  |
| Acenaphthene | mg/kg dw | < 10 | < 10 | < 10 | < 10 |  |
| Acenaphthylene | mg/kg dw | < 10 | < 10 | < 10 | < 10 |  |
| Aliphatics C5-C8 | mg/kg dw | < 10 | < 10 | < 10 | < 10 |  |
| Antimony | mg/kg dw | < 10 | < 10 | < 10 | < 10 | 30 |
| Anthracene | mg/kg dw | < 10 | < 10 | < 10 | < 10 |  |
| Aromatic C9-C10 | mg/kg dw | < 10 | < 10 | < 10 | < 10 | 100 |
| Arsenic | mg/kg dw | 10.8 | < 10 | < 10 | < 10 | 50 |
| Benzene | mg/kg dw | < 1 | < 1 | < 1 | < 1 | 2 |
| Benzo(a)anthracene | mg/kg dw | < 10 | < 10 | < 10 | < 10 | 10 |
| Benzo(a)pyrene | mg/kg dw | < 10 | < 10 | < 10 | < 10 | 10 |
| Benzo(b)fluoranthene | mg/kg dw | < 10 | < 10 | < 10 | < 10 | 10 |
| Benzo(e)pyrene | mg/kg dw | < 10 | < 10 | < 10 | < 10 |  |
| Benzo(j)fluoranthene | mg/kg dw | < 10 | < 10 | < 10 | < 10 |  |
| Benzo(k)fluoranthene | mg/kg dw | < 10 | < 10 | < 10 | < 10 | 10 |
| Beryllium | mg/kg dw | < 10 | < 10 | < 10 | < 10 | 10 |
| Cadmium | mg/kg dw | < 10 | < 10 | < 10 | < 10 | 15 |
| Cobalt | mg/kg dw | < 10 | < 10 | < 10 | < 10 | 250 |
| Chrysene | mg/kg dw | < 10 | < 10 | < 10 | < 10 | 50 |
| Chrome | mg/kg dw | 18.5 | 16.75 | 12.35 | 17.2 | 800 |
| Dibenzo(a,e)pyrene | mg/kg dw | < 10 | < 10 | < 10 | < 10 | 10 |
| Dibenzo(a,h)anthracene | mg/kg dw | < 10 | < 10 | < 10 | < 10 | 10 |
| Dibenzo(a,h)pyrene | mg/kg dw | < 10 | < 10 | < 10 | < 10 | 10 |
| Dibenzo(a,i)pyrene | mg/kg dw | < 10 | < 10 | < 10 | < 10 | 10 |
| Dibenzo(a,l)pyrene | mg/kg dw | < 10 | < 10 | < 10 | < 10 | 10 |
| Dipentene | mg/kg dw | < 1 | < 1 | < 1 | < 1 |  |
| Ethylbenzene | mg/kg dw | < 1 | < 1 | < 1 | < 1 | 50 |
| Phenanthrene | mg/kg dw | < 10 | < 10 | < 10 | < 10 |  |
| Fluoranthene | mg/kg dw | < 10 | < 10 | < 10 | < 10 |  |
| Fluorene | mg/kg dw | < 10 | < 10 | < 10 | < 10 |  |
| Indeno(1,2,3-c,d)pyrene | mg/kg dw | < 10 | < 10 | < 10 | < 10 |  |
| Isopropylbenzene (Cumene) | mg/kg dw | < 1 | < 1 | < 1 | < 1 |  |
| Mercury | mg/kg dw | < 1 | < 1 | < 1 | < 1 |  |
| Molybdenum | mg/kg dw | < 10 | < 10 | < 10 | < 10 |  |
| Naphthalene | mg/kg dw | < 10 | < 10 | < 10 | < 10 |  |
| Nickel | mg/kg dw | 19.95 | 15.1 | 12 | 16.1 | 500 |
| Lead | mg/kg dw | 62.3 | 50.35 | 40.2 | 43.15 | 1000 |
| Pyrene | mg/kg dw | < 10 | < 10 | < 10 | < 10 | 50 |
| Copper | mg/kg dw | 40.35 | 36.25 | 28.35 | 29.75 | 600 |
| Selenium | mg/kg dw | < 10 | < 10 | < 10 | < 10 | 15 |
| Pond | mg/kg dw | < 10 | < 10 | < 10 | < 10 | 350 |
| Styrene | mg/kg dw | < 1 | < 1 | < 1 | < 1 | 50 |
| Thallium | mg/kg dw | < 10 | < 10 | < 10 | < 10 | 10 |
| Toluene | mg/kg dw | < 1 | < 1 | < 1 | < 1 | 50 |
| Vanadium | mg/kg dw | 73.55 | 57.8 | 46.35 | 49.1 | 250 |
| Xylene | mg/kg dw | < 1 | < 1 | < 1 | < 1 | 50 |
| Zinc | mg/kg dw | 244.5 | 248 | 182.5 | 179.5 | 1500 |

Table 5 S.M. Parameters analysed on the leached obtained through the leaching test performed at the end of the experiment on the treated soil. The reported limits referred to the Italian DM 186/2006 Table 3 and D.Lgs. 152/2006: part IV, Title V, annex 5- Table 2.

| **Analyte** | **Unit of measurement** | **C** | **BA** | **BS** | **BAS** | **Limits** |
| --- | --- | --- | --- | --- | --- | --- |
| Nitrates | mg/L NO3 | < 0.5 | < 0.5 | 101.75 | 170.9 | 50 |
| Fluorides | mg/L | 0.407 | 0.4135 | 0.1111 | 0.12895 | 1.5 |
| Sulphates | mg/L SO4 | 30.3 | 27.35 | 11.85 | 18.95 | 250 |
| Chlorides | mg/L | 6.1 | 8.65 | < 2.5 | 8.9 | 100 |
| Cyanides | µg/L | < 10 | < 10 | < 10 | < 10 | 50 |
| Barium | mg/L | 0.00261 | 0.003045 | 0.00317 | 0.00563 | 1 |
| Copper | mg/L | 0.005445 | 0.00135 | 0.0092 | 0.010695 | 0.05 |
| Zinc | mg/L | 0.00327 | 0.001225 | 0.0062 | 0.00722 | 3 |
| Beryllium | µg/L | < 0.1 | < 0.1 | < 0.1 | < 0.1 | 10 |
| Cobalt | µg/L | < 1 | < 1 | < 1 | 1.25 | 250 |
| Nickel | µg/L | 1.7 | 1.75 | 6.25 | 5.65 | 10 |
| Vanadium | µg/L | 32.35 | 28.65 | 32.8 | 30.85 | 250 |
| Arsenic | µg/L | 6.5 | 10.5 | 25.2 | 21.95 | 50 |
| Cadmium | µg/L | < 0.5 | < 0.5 | < 0.5 | < 0.5 | 5 |
| Total Chrome | µg/L | 2.45 | 4.1 | 4.1 | < 1 | 50 |
| Lead | µg/L | < 1 | < 1 | < 1 | < 1 | 50 |
| Selenium | µg/L | < 1 | < 1 | < 1 | < 1 | 10 |
| Mercury | µg/L | < 0.1 | < 0.1 | < 0.1 | < 0.1 | 1 |
| Asbestos | mg/L | < 0.5 | < 0.5 | < 0.5 | < 0.5 | 30 |
| Chemical oxygen demand (COD) | mg/L O2 | 71.65 | 124.5 | 73.35 | 72.75 | 30 |
| Benzene | µg/L | < 0.1 | < 0.1 | < 0.1 | < 0.1 | 1 |
| p-Xylene | µg/L | < 1.0 | < 1.0 | < 1.0 | < 1.0 | 10 |
| Ethylbenzene | µg/L | < 1.0 | < 1.0 | < 1.0 | < 1.0 | 50 |
| Styrene | µg/L | < 1.0 | < 1.0 | < 1.0 | < 1.0 | 25 |
| Toluene | µg/L | < 1.0 | < 1.0 | < 1.0 | < 1.0 | 15 |

Figure 1 S.M. Comparison among the chromatograms of samples collected at the beginning of the experiment (t0), at the end of the Rapid phase (t8) and at the end of Slow phase (t16) in tests C, BS and BAS.

Table 6 S.M. Assessment of the statical differences between the PCA results: p-value obtained by the pairwise comparisons using PERMANOVA on a Euclidean distance matrix.

|  | **C** | **BA** | **BS** |
| --- | --- | --- | --- |
| **BA** | 0.0012 | - | - |
| **BS** | 0.0012 | 0.0012 | - |
| **BAS** | 0.0012 | 0.0012 | 0.0949 |

Figure 2 S.M. Average concentration time-profile of Ptot, Ntot and TOC.

Figure 3 S.M. Average concentration time-profile of aerobic CFU, anaerobic CFU, pH and T.
